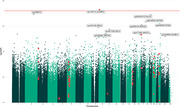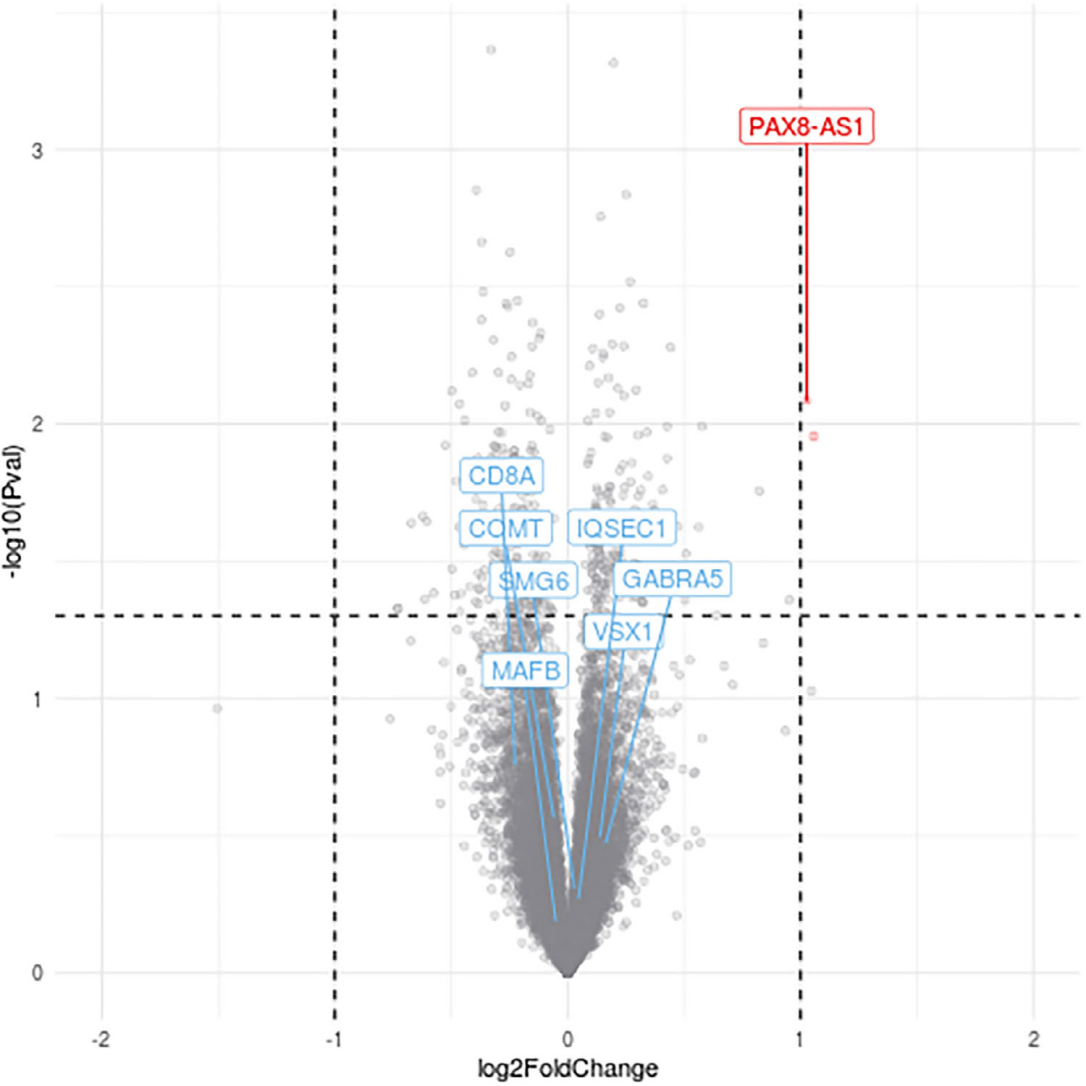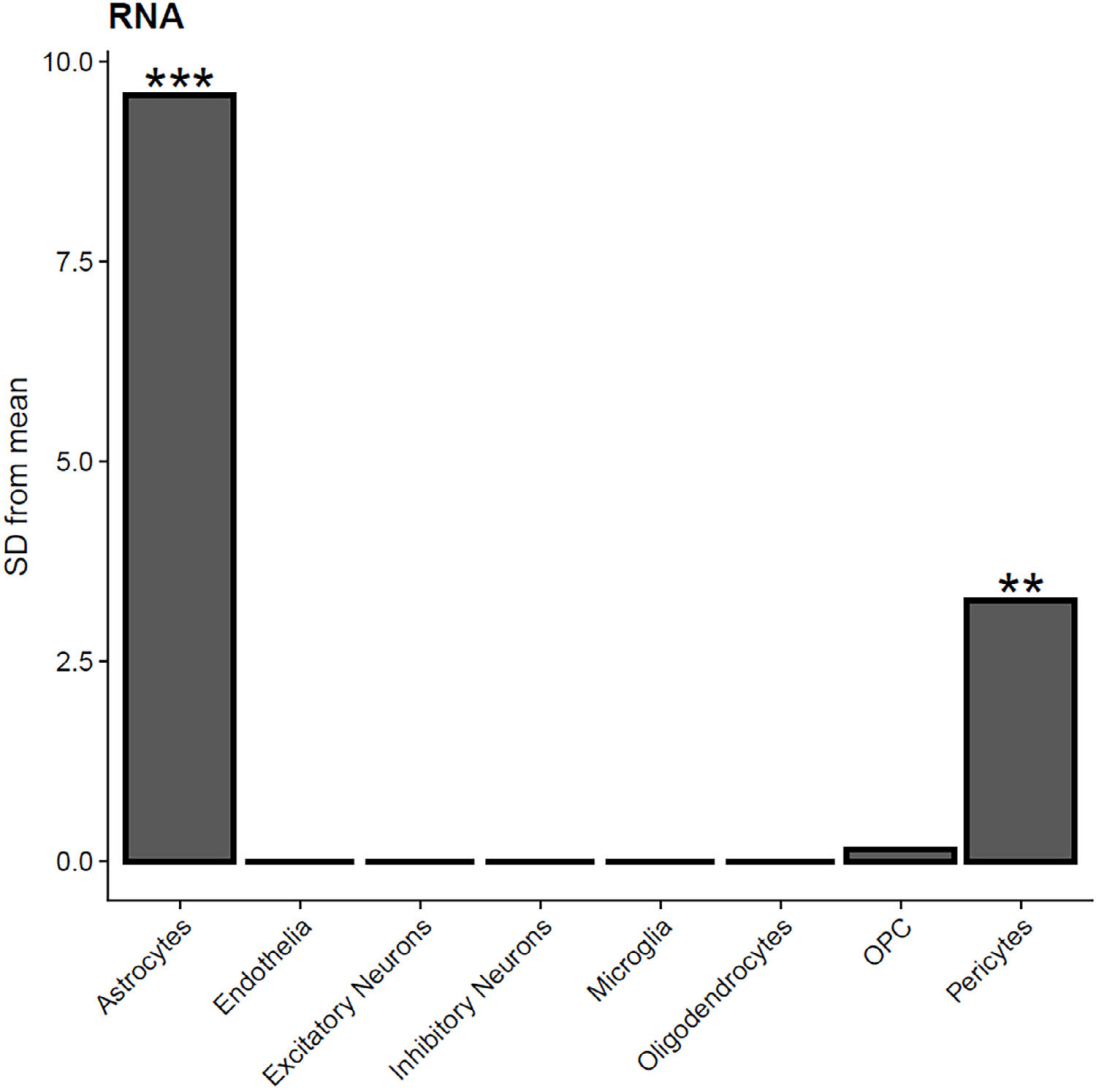# Distinct Methylomic Signatures Emerge in the Prefrontal Cortex Following Amyloid‐Beta Immunisation, with Altered Expression Patterns in Astrocytes

**DOI:** 10.1002/alz.091497

**Published:** 2025-01-03

**Authors:** Lachlan Ford MacBean, Adam Smith, Delphine Boche, Katie Lunnon

**Affiliations:** ^1^ University of Exeter, Exeter, Devon United Kingdom; ^2^ University of Exeter, Exeter United Kingdom; ^3^ University of Southampton, Southampton United Kingdom

## Abstract

**Background:**

Post‐mortem neuropathological examinations following the first active immunotherapy strategy (AN‐1792, Elan Pharmaceuticals, 2000) for Alzheimer’s disease (AD) have evidenced amyloid‐β (Aβ) plaque clearance and increased microglial phagocytic activity in immunised individuals. This study characterises the epigenetic profiles of individuals who underwent Aβ immunotherapy with the aim of discovering novel therapeutic targets and biomarkers.

**Method:**

DNA and RNA was isolated from post‐mortem prefrontal cortex tissue of immunised cases (n = 14) who received varying doses (ug) and number of doses during the trial period. DNA methylation was quantified using methylation arrays and the raw intensity values processed and normalised for subsequent statistical analysis to identify differentially methylated positions (DMPs) across the genome associated with Aβ immunotherapy. Additional analyses included cell enrichment analysis of the top most differentially expressed genes.

**Result:**

After correcting for common known variables (age, sex, cell type composition) and batch effects, a DMP located within CUGBP Elav‐like family member 2 (*CUGBP2*) at the genome‐wide significance level (*P* < 9.00E−08) was associated with Aβ immunisation. 10 DMRs were further associated with immunisation, with nine regions consisting of ≥ 3 CpG sites and having a Sidak‐corrected *P* < 0.05. In addition, a similar model performed on the RNA‐sequencing data showed no overlap with the aforementioned DMRs, but did exhibit significant gene expression alterations associated with astrocytes.

**Conclusion:**

Indicating better inflammatory markers can help inform of effective preventative care strategies for elders. This study provides evidence for altered epigenetic processes in the pathophysiology of AD and identifies novel processes specific to Aβ immunotherapy. The next step for this project includes analysing genotyping data to identify potential methylation quantitative trait loci and further our understanding of the relationship between single nucleotide polymorphisms and methylation changes.